# Preserved peak exercise capacity in Andean highlanders with excessive erythrocytosis both before and after isovolumic hemodilution

**DOI:** 10.1152/japplphysiol.00439.2022

**Published:** 2022-11-23

**Authors:** Cecilia Anza-Ramírez, Wanjun Gu, José L. Macarlupú, Rómulo J. Figueroa-Mujíca, Gustavo A. Vizcardo-Galindo, Erica C. Heinrich, Michael S. Tift, Harrieth E. Wagner, Peter D. Wagner, Tatum S. Simonson, Francisco C. Villafuerte

**Affiliations:** ^1^Facultad de Ciencias y Filosofía, Laboratorio de Fisiología Comparada/Laboratorio de Fisiología del Transporte de Oxígeno, Universidad Peruana Cayetano Heredia, Lima, Perú; ^2^Division of Pulmonary, Critical Care, and Sleep Medicine, School of Medicine, University of California San Diego, La Jolla, California; ^3^Division of Biomedical Sciences, School of Medicine, University of California Riverside, Riverside, California; ^4^Department of Biology and Marine Biology, University of North Carolina at Wilmington, Wilmington, North Carolina

**Keywords:** Andean highlanders, chronic mountain sickness, exercise capacity, excessive erythrocytosis, isovolumic hemodilution

## Abstract

In chronic mountain sickness (CMS), increased blood oxygen (O_2_)-carrying capacity due to excessive erythrocytosis (EE, [Hb] ≥ 21 g/dL) could be offset, especially during exercise by both impaired cardiac output (Q̇t) and O_2_ diffusion limitation in lungs and muscle. We hypothesized that EE results in reduced peak V̇o_2_ despite increased blood O_2_-carrying capacity, and that isovolumic hemodilution (IVHD) improves exercise capacity. In 14 male residents of Cerro de Pasco, Peru (4,340 m), six with and eight without EE, we measured peak cycle-exercise capacity, V̇o_2_, Q̇t, arterial blood gas parameters, and (resting) blood volume. This was repeated for participants with EE after IVHD, reducing hematocrit by 20% (from 67% to 53%). From these data, we quantified the major O_2_ transport pathway components (ventilation, pulmonary alveolar-capillary diffusion, Q̇t, and blood-muscle mitochondria diffusion). Participants with EE had similar peak V̇o_2_, systemic O_2_ delivery, and O_2_ extraction as non-EE controls, however, with lower Q̇t and higher arterial [O_2_]. After IVHD, peak V̇o_2_ was preserved (but not enhanced), with lower O_2_ delivery (despite higher Q̇t) balanced by greater O_2_ extraction. The considerable variance in exercise capacity across the 14 individuals was explained essentially completely by differences in both pulmonary and muscle O_2_ diffusional conductances and not by any differences in ventilation, [Hb], nor Q̇t. In conclusion, EE does not result in lower peak V̇o_2_ in Andean males, and IVHD maintains, but does not enhance, exercise capacity.

**NEW & NOTEWORTHY** Male Andean highlanders with and without excessive erythrocytosis (EE) have similar peak V̇o_2_ at 4,340 m, with higher arterial [O_2_] in EE and lower cardiac output (Q̇t), thus maintaining similar O_2_ delivery. Peak V̇o_2_ in participants with EE was unaffected by isovolumic hemodilution (hematocrit reduced from 67% to 53%), with lower O_2_ delivery balanced by slightly increased Q̇t and greater O_2_ extraction. Differences in lung and muscle diffusing capacity, and not hematocrit variation, accounted for essentially all interindividual variance in peak V̇o_2_.

## Graphical abstract

**Figure:**
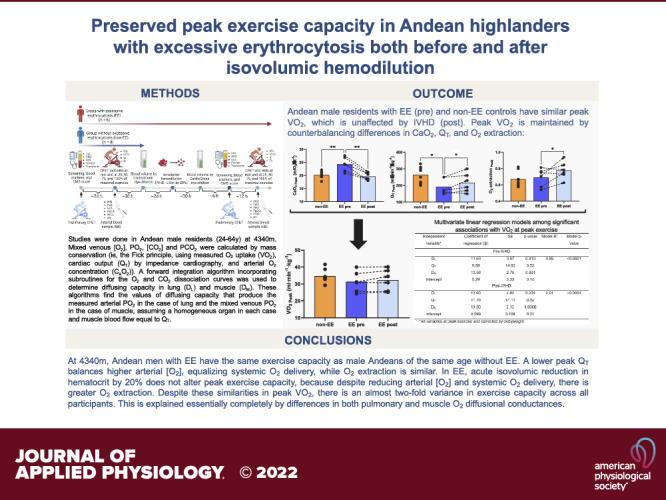

## INTRODUCTION

Human populations have inhabited high-altitude regions for thousands of years and exhibit different strategies to cope with life under hypoxic conditions (1–3). Relative to Tibetan and Ethiopian highlanders at comparable altitudes, Andeans show slightly higher average hemoglobin concentration ([Hb]) (1, 4) and many develop excessive erythrocytosis (EE; [Hb] ≥ 21 g/dL in men and ≥19 g/dL in women) and chronic mountain sickness (CMS, also called Monge’s disease). CMS is a maladaptive clinical syndrome characterized by the presence of EE accompanied by headache, shortness of breath and/or palpitations, sleep disturbance, cyanosis, paresthesia, widespread dilatation of veins, and tinnitus (5, 6). The condition is also frequently associated with pulmonary hypertension (7) and adverse cardiometabolic risk factors (8–14). Chronic hypoxemia is considered as the main underlying cause of EE and CMS, and, although the pathophysiological mechanisms are controversial, there is a clear underlying cellular and genetic basis for the exaggerated erythrocyte production (15–21).

Travel to lower altitudes, bloodletting (blood withdrawal without fluid replacement), and hemodilution (blood withdrawal with fluid replacement) are common temporary treatments of CMS signs and symptoms, suggesting a significant role for EE in the development of the syndrome (22–24). Isovolumic hemodilution (IVHD) involves the removal of a specified volume of blood with immediate replacement by the same volume of a colloidal solution such as albumin, dextran, hydroxyethyl starch, or degraded gelatin derivatives. The few studies using IVHD in highlanders with CMS have shown varying results in terms of arterial O_2_ saturation, pulmonary ventilation, hemodynamics, exercise capacity (23–25), in addition to increased cardiac output and ventilation/perfusion (V̇a/Q̇) mismatch (25). Smith et al. (26) showed that the reduction of hematocrit by 19% through IVHD (∼500 mL of blood/day over 4 days replaced with normal saline, 0.9% NaCl) in highlanders with CMS caused a 10% increase in resting cardiac output, an increase in pulmonary artery systolic pressure (PASP), in addition to a progressive development of iron deficiency accompanied by a further significant increase in PASP over 12 days post-IVHD. Moreover, IVHD in highlanders with CMS significantly reduced blood viscosity and improved shear stress stimulus-adjusted flow-mediated dilation, suggesting that the resistance to flow incurred by blood viscosity impairs shear stress-associated vasodilation, impairing perfusion and potentially impairing regional O_2_ delivery to tissues (27). However, none of these recent studies examined the effect of excessive hematocrit and hemodilution on aerobic capacity and overall O_2_ transport.

Previous studies in Andean (23, 28) and Tibetan (23, 28) highlanders revealed an inverse relationship between maximal exercise capacity and [Hb], and recent studies provide evidence for a positive association between Hb mass and V̇o_2peak_ in Sherpa (29). A later study in Andeans with [Hb] ranging from standard high-altitude to excessive values at 4,340 m showed similar aerobic capacities and no relationship between [Hb] and V̇o_2max_ (30). However, the reference group for this study was not well matched with the patients with CMS. It included male and female highlanders with lower V̇o_2max_ and lower hematocrit and individuals who were ∼10 yr younger in age than the group with CMS. Finally, a more recent study has also shown a lack of association between [Hb] and V̇o_2max_ in male Andean highlanders (31). Excessively elevated hematocrit might reduce tissue O_2_ delivery due to impaired diffusion and offset the advantage of increased O_2_ carrying capacity due to high [Hb]. As shown 40 years ago by Piiper and Scheid (32), an elevated [Hb] may contribute to diffusion limitation of O_2_ exchange. This limitation could occur both between alveolar gas and capillary blood in the lungs and between muscle microcirculatory vessels and mitochondria (33), whereby the compound term D/(β·Q̇) determines the degree of diffusion equilibration to be expected (D is lung or muscle diffusing capacity, β is the average slope of the O_2_-Hb dissociation curve, and Q̇ is blood flow). Since elevated [Hb] directly increases β, D/(β·Q̇) will be reduced for given values of D and Q̇, potentially causing O_2_ diffusion limitation. These O_2_ diffusional transport steps, together with the capacity for ventilatory and circulatory convective O_2_ transport, define how the O_2_ transport pathway as a system determines maximal aerobic capacity or V̇o_2max_ (34). Therefore, the study of maximal exercise capacity can provide fundamental insight into the effects of an elevated [Hb], and EE in particular, on each of the steps in O_2_ transport from inspired air to muscle mitochondria.

We hypothesized that despite high blood O_2_-carrying capacity, individuals with EE, relative to those without, would have lower exercise capacity that would increase following IVHD. Therefore, the primary aim of the present study was to examine the effect of EE on peak V̇o_2_ in Andean men resident in Cerro de Pasco, Peru, by making measurements in *1*) healthy participants without EE, *2*) participants with EE the day before IVHD, and *3*) the same EE participants 48 h following IVHD designed to reduce hematocrit by 20%. As a secondary aim, we assessed each major convective and diffusive step of the O_2_ transport pathway (ventilation, alveolar-capillary diffusion, cardiac output, and blood-mitochondria diffusion) to provide insight at an integrated physiological systems level that will guide future mechanistic studies.

## MATERIALS AND METHODS

### Ethical Approval

The study was approved by the Institutional Ethics Committee of Universidad Peruana Cayetano Heredia (CIEH-UPCH 081-03-17, SIDISI #59285) and by the University of California, San Diego Human Research Protection Program (UC San Diego Project #171772). All participants received a detailed explanation of the study procedures and signed an informed consent form in Spanish.

### Study Participants

Fourteen volunteers, six with EE and eight age-matched highlanders without EE, participated in the study. All participants were nonathletic but active men between 24 and 64 yr old and lifelong residents of Cerro de Pasco, Peru (4,340 m). Only male highlanders were included in the study because EE and CMS have a very low prevalence in premenopausal women (35, 36). Therefore, forming comparable age-matched groups without any confounding effects of age and menopause itself was not possible. The inclusion of only male participants means that our results cannot be generalized to women nor to males outside the age range studied.

No participants had a history of pulmonary, cardiovascular, or renal disease, were current smokers, worked in mining activities, had undergone blood transfusions or phlebotomies in the previous 6 mo, had traveled to lower altitudes (<3,000 m) for more than 7 days during the previous 6 mo, or had demonstrated abnormal ECG or pulmonary function during screening procedures.

### Preliminary Screening, Hematocrit, and Qinghai CMS Score

Clinical examination was performed during a preliminary screening session to rule out prior history of cardiovascular or pulmonary disease. During this session, an ECG (Quark C12x, Cosmed, Italy) and spirometry test (Pony FX, Cosmed, Italy) were performed, and pulse oxygen saturation ($Sp_{O_{2}}$) and heart rate (HR) were measured using a Nellcor N-560 oximeter (Nellcor Puritan Bennet Inc., Pleasanton CA). Systolic and diastolic blood pressure (SBP and DBP, respectively) were measured with a validated oscillometric device [UA-767Plus, A&D, Japan (37)]. All measurements were office-based taken after a 5-min resting period.

For screening purposes, hematocrit was determined from duplicate centrifuged micro blood samples obtained from fingertip capillary blood. Participants with hematocrit ≥ 63% (equivalent to [Hb] ≥ 21 g/dL) were classified as individuals with EE (5). General health and Qinghai CMS score questionnaires were also applied (Table 1). The CMS score quantifies the absence or presence and severity of the syndrome and is based on the occurrence of EE, as well as the presence and severity of the following signs and symptoms: headache, shortness of breath or palpitations, sleep disturbances, paresthesia, cyanosis, dilated veins, and tinnitus (5).

**Table 1.** Baseline characteristics of study participants

	Non-EE (*n* = 8)	EE Pre-IVHD (*n* = 6)	*P* Value
Age, yr	52 ± 9	50 ± 14	0.69
Bodyweight, kg	64 ± 7	70 ± 16	0.40
BMI, kg/m^2^	24.8 ± 2.4	25.2 ± 4.5	0.83
Hematocrit, %	55 ± 2	69 ± 3	0.0001***
CMS score	1.1 ± 1.0	10.0 ± 5.5	0.003**
HR, beats/min	68 ± 5	69 ± 10	0.82
SBP, mmHg	102 ± 12	107 ± 10	0.42
DBP, mmHg	68 ± 10	68 ± 12	0.99

Values expressed as means ± SD. BMI, body mass index; CMS, chronic mountain sickness; DBP, diastolic blood pressure; EE, excessive erythrocytosis; HR, heart rate; SBP, systolic blood pressure. Bold type indicates statistical significance at **P < 0.01, ***P < 0.001.

### Blood Samples for [Hb], Hematocrit, and Iron Profile

Two 9-mL blood samples were taken from the antecubital vein of all participants under fasting conditions on the morning of the preliminary cardiopulmonary exercise test (CPET). One sample was collected in a clot-activator tube and the second in an EDTA-coated tube, from which a microcapillary sample was taken to determine venous hematocrit and [Hb] using an iSTAT-1 handheld blood analyzer (iSTAT, Abbott Point of Care Inc. Princeton, NJ). Samples were centrifuged at 3,500 rpm for 20 min. Serum was immediately stored at −20°C, and then at −80°C for later analysis of iron profile. Forty-eight hours after hemodilution, a second set of blood samples for the same analyses were obtained and CMS score was assessed in participants within the EE group. Iron profiles were determined because of the effect of IVHD-induced reduction in iron concentration on pulmonary vascular function (26), which in turn might affect cardiac function during exercise.

### Blood Volume Measurement

Blood volume (BV) was measured once in participants without EE and twice in participants with EE (∼24 h before and ∼30 h after IVHD) using the indocyanine green (ICG; CardioGreen, Sigma-Aldrich) dye dilution method (38). Briefly, with participants seated, a catheter was placed in the cephalic vein with two three-way connected stopcocks. Ten to fifteen minutes before beginning the BV measurement procedure, participants were accommodated in a semirecumbent position. To avoid dye-contamination of post-IVHD blood samples, one stopcock was used for injection of a volume of dye at a concentration of 2.5 mg/mL (0.05 mg/kg) and the second for blood sample collection just before and 5, 10, 15, and 20 min after dye injection. Each sample was centrifuged, plasma collected, and its absorbance read at 805 nm. A four-point calibration curve was obtained by adding increasing indocyanine green amounts to the plasma samples of each participant to obtain final concentrations of 0.85–3.3 µg/mL. Plasma volume for each participant was calculated by mass conservation, using absorbance values extrapolated linearly back to time of dye injection (t_0_). Briefly, after exponential fitting ([Abs = Abs_0_·e^−kt^) of the absorbance curve resulting from the respective measuring time points, and posterior logarithmic transformation, the absorbance of ICG in plasma at t_0_ is obtained. After introducing this value into the calibration equation, we obtained [ICG] at t_0_ ([ICG]_0_). Knowing the ICG injected mass, we calculated PV as: PV = [ICG]_0_/ICG mass. Then, we calculated BV as: BV = PV/(1−hematocrit).

### Isovolumic Hemodilution

IVHD was performed to reduce hematocrit by ∼20% below its initial value. The volume of blood required to be removed to obtain this planned reduction in hematocrit was calculated using the methodology of Gross et al. (39):

$$\text{V}=\text{ BV}\times (\frac{{\text{Hct}}_{0}-{\text{Hct}}_{\text{F}}}{{\text{Hct}}_{\text{AV}}})$$

where V is the blood volume to be removed, BV is the total blood volume (measured as above before hemodilution), Hct_0_ is the hematocrit before hemodilution, Hct_F_ is the hematocrit at the end of the hemodilution (or the target hematocrit), and Hct_AV_ is the average of these two hematocrit values (Hct_0_ + Hct_F_)/2.

Up to four 500 mL units of blood were removed in supine position from the cephalic vein of the nondominant arm over two consecutive days to reach the target hematocrit value. Each day the removed blood volume was substituted by an equal volume of colloid plasma replacement (Polygelyne 3.5%, Hisocel). Polygeline has a half-life of 3–6 h, and its hemodynamic stabilization effects last for 24 h (40, 41). Hematocrit was measured before each IVHD session to assess the need for additional blood volume removal. Each hemodilution session was conducted slowly (over ∼3 h) to avoid acute hemodynamic changes.

### Cardiopulmonary Exercise Test

Participants first performed a standard, noninvasive maximal CPET to exhaustion at the Universidad Peruana Cayetano Heredia high-altitude laboratory in Cerro de Pasco, Peru (4,340 m), according to the guidelines of the American Thoracic Society/American College of Chest Physicians (42). A forehead oximeter (Nellcor N-395, Louisville, KY) probe was taped to the skin to obtain $Sp_{O_{2}}$ measurements throughout the test. A tightly fitting silicone oro-nasal facemask (V2 series 7450 TM, Hans Rudolph, Kansas) was secured and connected to an ergospirometer-metabolic system (Quark CPET, Cosmed, Italy). CPET was performed on a cycle ergometer (Ergomedic 828E, Monark Exercise AB, Sweden) with continuous breath-by-breath (averaged every five breaths) measurements of respiratory parameters to determine O_2_ uptake (V̇o_2_), CO_2_ output (V̇co_2_), minute ventilation (V̇e), and end-tidal Po_2_ and Pco_2_ ($\text{P}E{\text{T}}_{{\text{O}}_{2}}$ and $\text{P}E{\text{T}}_{\text{C}{\text{O}}_{2}}$, respectively). Twelve-lead ECG and HR were recorded continuously.

In a second visit ∼24 h later, and after a 5-min rest while seated in the cycle ergometer, a five-step (3 min each) CPET was performed at power outputs corresponding to 0%, 25%, 50%, 75%, and 100% of each participant’s predetermined peak V̇o_2_. Before this five-step CPET session, a 20-gauge radial arterial catheter was placed percutaneously under local anesthetic using sterile technique to obtain arterial blood samples at rest and during the last minute of each workload step for hematocrit, [Hb], arterial blood gas (ABG) analysis (iSTAT, Abbott Point of Care Inc. Princeton, NJ), and direct measurement of arterial saturation ($\text{S}{\text{a}}_{{\text{O}}_{2}}$; AVOXimeter 4000, Whole Blood CO-oximeter, Instrumentation Laboratory, Edison, NJ). Impedance cardiography (IC, PhysioFlow Enduro, Paris, France) was used to measure cardiac output (Q̇t) by means of six electrodes taped onto cleansed skin (two each on the back, neck, and chest). We included a comparison of our Q̇t measurements obtained by IC with data from the classic study by Astrand et al. using the dye-dilution technique (43) (see appendix).

The multistage CPET, again with arterial sampling and impedance cardiography, was repeated in participants with EE ∼48 h after their last IVHD session, encouraging participants to achieve higher power outputs than before IVHD. Body weight was recorded before each CPET session and oral temperature was recorded before and immediately after.

Q̇t response to exercise was characterized by both peak Q̇t and the slope of the Q̇t-V̇o_2_ relationship. Mixed venous [O_2_] ($\text{C}{\text{v}}_{{\text{O}}_{2}}$) and PO_2_ ($\text{P}{\text{v}}_{{\text{O}}_{2}}$), together with mixed venous [CO_2_] ($\text{C}{\text{v}}_{\text{C}{\text{O}}_{2}}$) and PCO_2_ ($\text{P}{\text{v}}_{\text{C}{\text{O}}_{2}}$), were calculated by mass conservation equations using the Fick principle and measured values of V̇o_2_, Q̇t, and arterial O_2_ concentration ($C{a_{O}}_{2}$). Standard P_50_ (P_50 STD_, i.e., corrected to Pco_2_ of 40 mmHg, pH of 7.40, and temperature of 37°C) was computed for each subject from their Po_2_-saturation relationship (44). Previously established FORTRAN programs using a forward integration algorithm (45) and incorporating the Kelman subroutines for the O_2_ and CO_2_ dissociation curves (46, 47) were used to determine diffusing capacity in lung (Dl) and muscle (Dm). These algorithms find the values of diffusing capacity in lung and muscle that produce the measured arterial Po_2_ in the case of lung and the ${\text{Pv}}_{{\text{O}}_{2}}$ (determined as described at the beginning of this paragraph) in the case of muscle, assuming a homogeneous organ in each case. Stroke volume (SV) was calculated as Q̇t divided by HR, convective O_2_ transport (i.e., O_2_ delivery, Q̇o_2_) as the product of Q̇t and $\text{C}{\text{a}}_{{\text{O}}_{2}}$, and O_2_ extraction determined from the ratio of whole body V̇o_2_ and Q̇o_2_, all using data at peak exercise.

### Analysis of O_2_ Transport Variables during Maximal Exercise

Individual O_2_ transport variables measured or calculated at maximal exercise included peak V̇o_2_ (mL·min^−1^·kg^−1^), minute ventilation V̇e (mL·min^−1^·kg^−1^), $\text{P}{\text{a}}_{{\text{O}}_{2}}$, $\text{S}{\text{a}}_{{\text{O}}_{2}}$, $\text{C}{\text{a}}_{{\text{O}}_{2}}$, Dl normalized to body weight (Dl·kg^−1^), alveolar-arterial Po_2_ (A-aPo_2_) difference [obtained from the difference of $\text{P}{\text{A}}_{{\text{O}}_{2}}$ calculated by the alveolar gas equation ($\text{P}{\text{A}}_{{\text{O}}_{2}}$= $\text{P}{\text{I}}_{{\text{O}}_{2}}$ − $\text{P}{\text{a}}_{{\text{CO}}_{2}}$/R + $\text{P}{\text{a}}_{{\text{CO}}_{2}}$ × $\text{F}{\text{I}}_{{\text{O}}_{2}}$ × (1−R)/R) and $\text{P}{\text{a}}_{{\text{O}}_{2}}$], Q̇t (mL·min^−1^·kg^−1^), Q̇o_2_, ${\text{Pv}}_{{\text{O}}_{2}}$, mixed venous O_2_ saturation ($S{v_{O}}_{2}$), $\text{C}{\text{v}}_{{\text{O}}_{2}}$, D_M_ normalized to body weight (D_M_·kg^−1^), O_2_ extraction, $\text{P}{\text{a}}_{{\text{CO}}_{2}}$, arterial pH (pH_a_), $\text{P}{\text{v}}_{\text{C}{\text{O}}_{2}}$, mixed venous pH (pH_v_), arterial [lactate], and base excess (BE). These were each compared across groups. Next, peak V̇o_2_was examined as a function of individual O_2_ transport variables using linear regression, and significant associations were included into a multivariate linear regression model. Individual O_2_ transport variables were also examined as a function of arterial [Hb].

### Sample Size and Statistical Analysis

Assuming comparable differences and corresponding effect sizes (η^2^ = 1.6) previously observed in the effect of IVHD on exercise capacity in Andean highlanders with CMS (23, 24), our primary end-outcome variable (peak V̇o_2_) required a sample size of six participants to achieve a power of 0.80 at *P* = 0.02.

Normality of distribution and homogeneity of variance were assessed for comparison between groups. Unpaired or paired Student’s or Wilcoxon tests were applied accordingly to evaluate differences between *1*) participants without EE and those with EE pre-IVHD, *2*) participants without EE and those with EE post-IVHD, and *3*) participants with EE pre- and post-IVHD. Bonferroni’s correction for multiple comparisons was applied to account for the dual use of each group (i.e., participants without EE compared with both participants with EE pre- and post-IVHD; participants with EE pre- and post-IVHD compared with each other) of the primary variable. As a result, α was set at 0.025. No correction was used for exploratory (secondary) variables.

Multiple linear regression was used to assess the significance of standardized predictors of peak V̇o_2_ using O_2_ transport pathway components as the independent variables. Interaction effects were further assessed for all significant predictors. GraphPad Prism v. 9.1.2 for Windows (GraphPad Software, San Diego, CA) was used for all statistical comparisons. Values are presented as means ± SD throughout the manuscript.

## RESULTS

### General Screening of Participants

Table 1 shows the general characteristics of study participants. Age, body weight, and BMI were similar between participants with and without EE, with a similar distribution of individual values between the groups. As expected, before IVHD, the six participants with EE (EE pre-IVHD) exhibited significantly higher hematocrit, and all but one had an elevated CMS score. Office-based measurements of HR, SBP, and DBP were similar between groups.

### Blood Volume and Isovolumic Hemodilution

**Figure 1.**
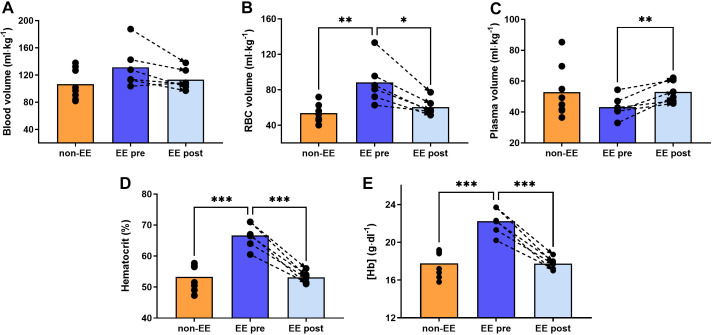
Blood volume, hemoglobin, and hematocrit. Comparison of blood volume (BV), red blood cell (RBC) total volume, plasma volume (PV), hematocrit, and hemoglobin concentration ([Hb]) in participants with and without EE before and after IVHD. Participants with EE tended to have larger BV before IVHD which was maintained after IVHD (*A*). Total RBC volume was larger in participants with EE and was reduced to non-EE levels after IVHD (*B*). *C* shows similar PV in highlanders with and without EE before IVHD. After IVHD, PV increased slightly in the EE group. *D* and *E* shows hematocrit and [Hb], respectively. As expected, both were significantly higher in the EE group and were reduced to non-EE levels after IVHD. The graphs show individual values, means represented as bars, and connecting discontinuous arrows indicate paired pre- and post-IVHD measurements. Differences between groups were assessed using unpaired Student’s *t* test to compare non-EE vs. EE pre-IVHD and EE post-IVHD, and a paired *t* test to compare EE pre- vs. post-IVHD. *P < 0.05, **P < 0.01, ***P < 0.001. All male participants, non-EE (*n* = 8), EE (*n* = 6). EE, excessive erythrocytosis; IVHD, isovolumic hemodilution.

Pre-IVHD BV was not different in participants with EE compared with participants without (131 ± 31 vs. 106 ± 22 mL/kg, respectively, *P* = 0.10, Fig. 1*A*). Forty-eight hours after removing ∼2–4 blood units (1,675 ± 442 mL), and replacing it with an equal volume of the plasma substitute, BV remained statistically not different to pre-IVHD values with a tendency to a 14% reduction (131 ± 31 to 113 ± 16 mL/kg, *P* = 0.07, Fig. 1*A*). Red blood cell (RBC) total volume was larger in the EE pre-IVHD group compared with the non-EE group, and decreased significantly post-IVHD (Fig. 1*B*). Plasma volume (PV) was not different between groups before IVHD and increased after IVHD (Fig. 1*C*; *P* = 0.006). As planned, a 20.1 ± 1.6% reduction of hematocrit (Fig. 1*D*) and [Hb] was achieved (Fig. 1, *D* and *E*, respectively, and Table 2). Bodyweight, BMI, and CMS score also decreased posthemodilution (−2.4 kg, *P* = 0.008; −0.85 kg/m^2^, *P* = 0.006; and −8.8 points, *P* = 0.017, respectively). Serum iron and ferritin were not different between groups before and decreased significantly post-IVHD (112.3 ± 46.7 vs. 60.8 ± 30.2 µg/dL, *P* < 0.01; 101.9 ± 122.4 vs. 52.4 ± 49.0 ng/mL, respectively).

**Table 2.** Participants with and without EE before and after IVHD under resting conditions

	Non-EE	EE Pre-IVHD	EE Post-IVHD	Non-EE vs. EE Pre-IVHD	EE Pre-IVH vs. EE Post-IVHD	Non-EE vs. EE Post-IVHD
Hct_a_, %	53 ± 4	67 ± 4	53 ± 2	0.0001***	0.0002***	0.95
[Hb]_a_, g·dL^-1^	17.8 ± 1.4	22.2 ± 1.4	17.7 ± 0.6	0.0001***	0.0002***	0.98
P_50 STD_, mmHg	25.9 ± 1.0	24.5 ± 1.1	26.3 ± 0.7	0.021*	0.007**	0.48
V̇o_2_, mL·min^−1^	326 ± 54	322 ± 61	300 ± 52	0.45	0.080	0.20
V̇o_2_, mL·min^−1^·kg^−1^	5.1 ± 0.6	4.8 ± 1.3	4.6 ± 0.8	0.61	0.38	0.22
Q̇t, L·min^−1^	5.6 ± 1.3	5.4 ± 0.9	5.2 ± 0.9	0.80	0.60	0.55
Q̇t, mL·min^−1^·kg^−1^	86.6 ± 15.2	79.2 ± 12.1	79.3 ± 17.1	0.35	0.99	0.41
HR, beats/min	86 ± 14	85 ± 8	80 ± 7	0.86	0.14	0.32
SV, mL·kg^−1^	1.02 ± 0.18	0.95 ± 0.20	0.99 ± 0.18	0.50	0.42	0.84
V̇e, L·min^−1^	11.8 ± 2.5	11.8 ± 2.2	11.4 ± 2.3	0.96	0.60	0.71
V̇e, mL·min^−1^·kg^−1^	184.8 ± 36.8	174.7 ± 41.9	170.5 ± 24.8	0.64	0.74	0.43
RER	0.87 ± 0.10	0.90 ± 0.08	0.90 ± 0.04	0.30	0.35	0.54
$\text{P}{\text{a}}_{{\text{O}}_{2}}$, mmHg	48.6 ± 5.7	45.7 ± 4.6	46.3 ± 3.8	0.32	0.73	0.41
$\text{S}{\text{a}}_{{\text{O}}_{2}}$, %	84 ± 4	79 ± 5	82 ± 5	0.030*	0.20	0.35
A-aPo_2_, mmHg	5.8 ± 2.5	9.1 ± 2.6	6.3 ± 1.2	0.033*	0.017*	0.67
$\text{P}{\text{a}}_{{\text{CO}}_{2}}$, mmHg	28.3 ± 4.9	29.0 ± 4.0	30.4 ± 2.2	0.69	0.42	0.27
pH_a_, units	7.39 ± 0.04	7.34 ± 0.01	7.38 ± 0.03	0.013*	0.026*	0.71
[HCO_3_^−^]_a_, mM	17.1 ± 3.0	15.6 ± 2.3	18.1 ± 0.8	0.33	0.046*	0.47
BE, mEq/L	−7.9 ± 3.3	−10.2 ± 2.3	−7.0 ± 0.9	0.17	0.029*	0.54
[Lactate]_a_, mM	0.90 ± 0.5	0.85 ± 0.3	0.49 ± 0.14	0.83	0.016*	0.071

A-aPo_2_, alveolar-arterial Po_2_ difference; BE, base excess; EE, excessive erythrocytosis; [HCO_3_^−^]_a_, arterial bicarbonate concentration; Hct_a_, arterial hematocrit; [Hb]_a_, arterial hemoglobin concentration; IVHD, isovolumic hemodilution; [Lactate]_a_: arterial lactate concentration; P_50 STD_, standard P50; $\text{P}{\text{a}}_{{\text{O}}_{2}}$, arterial Po_2_; $\text{P}{\text{a}}_{{\text{CO}}_{2}}$, arterial Po_2_; pH_a_, arterial pH; Q̇t, cardiac output; RER, respiratory exchange ratio; $\text{S}{\text{a}}_{{\text{O}}_{2}}$, arterial blood O_2_ saturation; V̇o_2_, O_2_ consumption; V̇e, pulmonary ventilation. Values expressed as means ± SD. Bold type indicates statistical significance at *P < 0.05, **P < 0.01, ***P < 0.001.

### Resting Conditions

Ambient conditions were stable with barometric pressure between 455 and 457 mmHg, room temperature between 14°C and 18°C, and relative humidity between 52% and 55%. Measurements were obtained after a 5-min resting period while the participant was seated in the cycle-ergometer. The principal data are shown in Table 2.

### Non-EE versus EE pre-IVHD

$\text{S}{\text{a}}_{{\text{O}}_{2}}$ was slightly lower, A-aPo_2_ larger, whereas P_50 STD_ and pH_a_ were slightly less in pre-IVHD participants with EE than in non-EE controls (*P* values marked in bold in the corresponding column of Table 2). All other parameters in Table 2 were not different between the control and pre-IVHD participants with EE.

### EE pre-IVHD versus EE post-IVHD

Post-IVHD, several parameters in Table 2 were slightly different from those before IVHD. $\text{S}{\text{a}}_{{\text{O}}_{2}}$, P_50 STD_, arterial bicarbonate concentration ([HCO_3_^−^]_a_), and pH_a_ were all higher, whereas A-aPo_2_, BE, and arterial lactate concentration ([lactate]_a_) were all lower, with all parameters therefore changing in the direction of normalization (*P* values marked in bold in the corresponding column of Table 2).

### EE post-IVHD versus non-EE

All parameters for post-IVHD participants with EE remained comparable or were not different from those in non-EE controls (Table 2).

### Peak Exercise

Oral temperature showed no significant differences before and after each CPET session with a maximum difference between 0.1°C and 0.3°C.

#### Non-EE versus EE pre-IVHD.

##### Peak V̇o_2_ and O_2_ transport from air to arterial blood.

**Figure 2.**
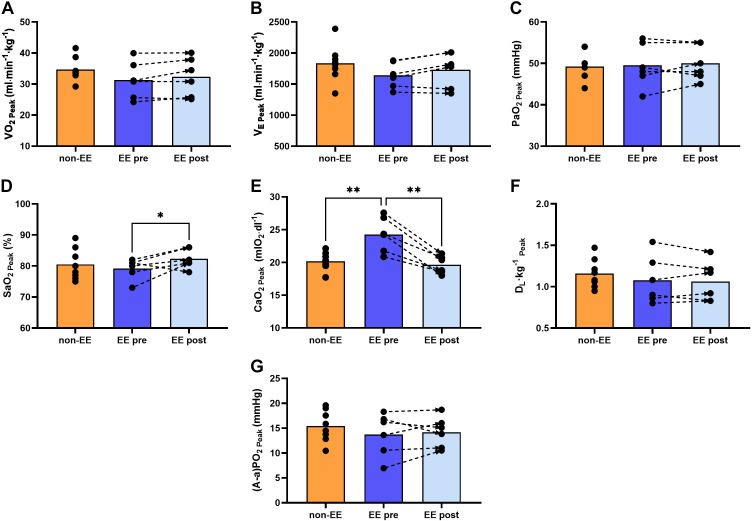
V̇o_2peak_ and O_2_ transport from air to arterial blood at peak exercise. Comparisons of V̇o_2_ and the different components of the O_2_ transport chain from air to arterial blood in highlanders with and without EE before and after IVHD at peak exercise. Peak V̇o_2_ (V̇o_2peak_) was similar between groups and remained unchanged after IVHD (*A*). Pulmonary ventilation at peak exercise (V̇e_Peak_) was similar between groups before IVHD (*B*). *C* shows similar arterial Po_2_ ($\text{P}{\text{a}}_{{\text{O}}_{2}}$) between groups and unchanged values after IVHD, whereas arterial O_2_ saturation ($Sa_{O_{2}}$) increased after IVHD as shown in *D*. *E* shows that arterial O_2_ content ($Ca_{O_{2}}$) was significantly higher in participants with EE compared with those without EE, and was markedly reduced after IVHD. Lung diffusion capacity (Dl) and alveolar-arterial Po_2_ difference A-aPo_2_ were similar between groups, and remained unchanged after IVHD as shown in *F* and *G*, respectively. The graphs show individual values, means represented as bars, and connecting discontinuous arrows indicate paired pre- and post-IVHD measurements. Differences between groups were assessed using unpaired Student’s *t* test to compare non-EE vs. EE pre-IVHD and EE post-IVHD and a paired test to compare EE pre- vs. post-IVHD *P < 0.05, **P < 0.01. All male participants, non-EE (*n* = 8), EE (*n* =6). EE, excessive erythrocytosis; IVHD, isovolumic hemodilution.

There were no differences between highlanders with and without EE in peak V̇o_2_ or V̇e (Fig. 2, *A* and *B*). $\text{P}{\text{a}}_{{\text{O}}_{2}}$ and $\text{S}{\text{a}}_{{\text{O}}_{2}}$ were also not different between groups (Fig. 2, *C* and *D*), whereas $\text{C}{\text{a}}_{{\text{O}}_{2}}$ was higher in the EE compared with the non-EE group (Fig. 2*E*). Dl and A-aPo_2_ were not different between groups (Fig. 2, *F* and *G*). Peak power output was also not different in non-EE and EE pre-IVHD groups (150.4 ± 13.4 vs. 138.0 ± 12.3 W, *P* = 0.10).

##### Peak Q̇t, SV, HR, and O_2_ transport from arterial blood to muscle.

**Figure 3.**
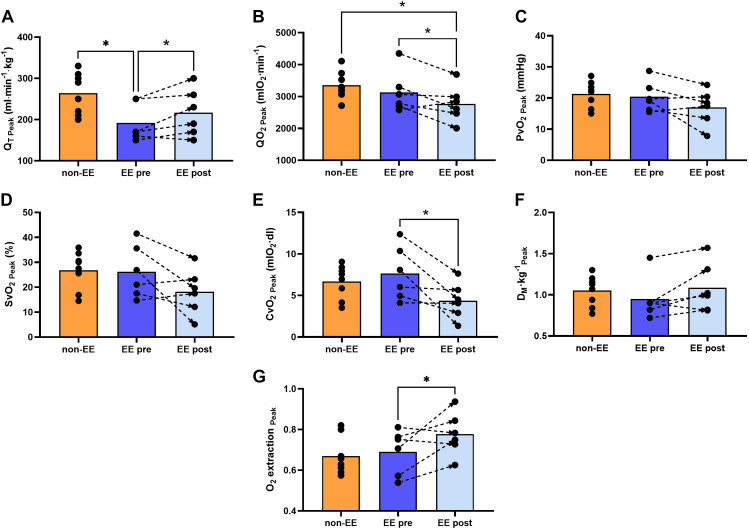
Q̇t_Peak_ and O_2_ transport from arterial blood to muscle at peak exercise. Comparisons of peak cardiac output (Q̇t_Peak_) and O_2_ transport from arterial blood to muscle between highlanders with and without EE before and after IVHD at peak exercise. Q̇t_Peak_ was lower in EE compared with non-EE highlanders before IVHD and increased to match non-EE levels after IVHD (*A*). Convective O_2_ transport or O_2_ delivery (Q̇o_2_) was similar between groups before IVHD and decreased after hemodilution but remained lower than non-EE controls (*B*). *C* and *D* show similar venous Po_2_ (${\text{Pv}}_{{\text{O}}_{2}}$) and venous O_2_ saturation ($Sv_{O_{2}}$) between groups and unchanged values after IVHD. *E* shows that venous O_2_ content ($\text{C}{\text{v}}_{{\text{O}}_{2}}$) was similar in EE compared with non-EE highlanders and was markedly reduced after IVHD. Muscle diffusing capacity (Dm) was similar between groups and remained unchanged after IVHD (*F*), whereas O_2_ extraction determined from the ratio of whole body V̇o_2_ and Q̇o_2_ was also similar between groups but increased after IVHD as shown in *G*. The graphs show individual values, means represented as bars, and connecting discontinuous arrows indicate paired pre- and post-IVHD measurements. Differences between groups were assessed using unpaired Student’s *t* test to compare non-EE vs. EE pre-IVHD and EE post-IVHD and a paired test to compare EE pre- vs. post-IVHD *P < 0.05. All male participants, non-EE (*n* = 8), EE (*n* = 6). EE, excessive erythrocytosis; IVHD, isovolumic hemodilution.

Peak Q̇t was lower in highlanders with EE before IVHD compared with the non-EE group (Fig. 3*A*) as a result of a slightly lower SV (1.3 ± 0.3 vs. 1.6 ± 0.3 mL/kg, *P* = 0.06). HR was not different in both groups (153 ± 19 vs. 165 ± 10 beats/min, respectively, *P* = 0.16), whereas peak Q̇o_2_, ${\text{Pv}}_{{\text{O}}_{2}}$, $\text{S}{\text{v}}_{O_{2}}$, $\text{C}{\text{v}}_{{\text{O}}_{2}}$, Dm, and O_2_ extraction were not different between groups (Fig. 3, *B–G*, respectively).

##### Acid-base status.

**Figure 4.**
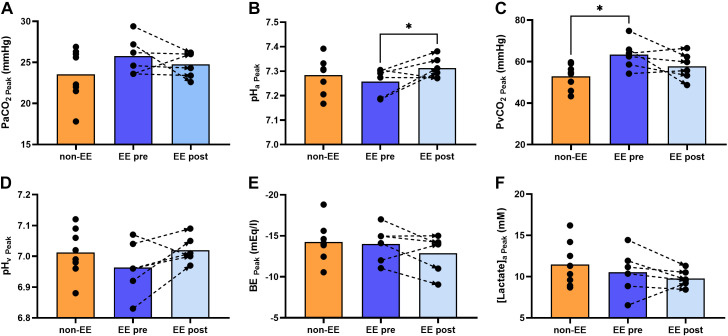
Acid-base status at peak exercise. Comparison of acid-base balance contributors in participants without and those with EE before and after IVHD at peak exercise. Arterial Pco_2_ at peak exercise ($\text{P}{\text{a}}_{{\text{CO}}_{2}}$_Peak_) was similar between groups before and after IVHD (*A*). Arterial pH (pH_a_) was similar between groups before IVHD and increased to non-EE levels after hemodilution (B). Venous Pco_2_ ($\text{P}{\text{v}}_{\text{C}{\text{O}}_{2}}$) was higher in the EE compared with the non-EE group and trended to decreased after IVHD (*C*), whereas venous pH (pHv) trended to lower values in EE before IVHD and trended to increase after (*D*). BE and [lactate]_a_ remained statistically similar across groups and unchanged after IVHD as shown in *E* and *F*, respectively. The graphs show individual values, means represented as bars, and connecting discontinuous arrows indicate paired pre- and post-IVHD measurements. Differences between groups were assessed using unpaired Student’s *t* test to compare non-EE vs. EE pre-IVHD and EE post-IVHD and a paired test to compare EE pre- vs. post-IVHD *P < 0.05. All male participants, non-EE (*n* = 8), EE (*n* = 6). BE, base excess; EE, excessive erythrocytosis; IVHD, isovolumic hemodilution.

$\text{P}{\text{a}}_{{\text{CO}}_{2}}$ and pH_a_ were not different between groups (Fig. 4, *A* and *B*, respectively), whereas $\text{P}{\text{v}}_{\text{C}{\text{O}}_{2}}$ was higher in the CMS group (Fig. 4*C*). pH_v_, [HCO_3_^−^]_a_, BE, and [lactate]_a_ were comparable between groups (Fig. 4, *D–F*, respectively).

#### EE pre-IVHD versus EE post-IVHD.

##### Peak V̇o_2_ and O_2_ transport from air to arterial blood.

Peak V̇o_2_ and V̇e remained essentially unchanged after hematocrit reduction (Fig. 3, *A* and *B*). $\text{P}{\text{a}}_{{\text{O}}_{2}}$ remained unchanged and $\text{S}{\text{a}}_{{\text{O}}_{2}}$ increased after IVHD (Fig. 3, *C* and *D*). As expected, $\text{C}{\text{a}}_{{\text{O}}_{2}}$ decreased significantly after IVHD (Fig. 2*E*). D_L_ and A-aPo_2_ did not change (Fig. 3, *F* and *G*). Peak power output was not different between EE pre- and EE post-IVHD groups (138.0 ± 12.3 vs. 140.5 ± 13.1 W, *P* = 0.36).

##### Peak Q̇t, SV, HR, and O_2_ transport from arterial blood to muscle.

Peak Q̇t increased slightly but significantly after IVHD (Fig. 3*A*). However, peak Q̇o_2_ decreased in EE post-IVHD (Fig. 3*B*) because of the larger reduction in [Hb] than rise in Q̇t. SV increased slightly (1.3 ± 0.3 vs. 1.4 ± 0.3 mL/kg, *P* = 0.13) and HR remained not different after IVHD (153 ± 19 vs. 155 ± 20 beats/min, *P* = 0.60). ${\text{Pv}}_{{\text{O}}_{2}}$ and $Sv_{O_{2}}$ at peak exercise showed a trend to decrease after IVHD (Fig. 3, *C* and *D*), whereas $\text{C}{\text{v}}_{{\text{O}}_{2}}$ decreased significantly (Fig. 3*E*). D_M_ remained unchanged, whereas O_2_ extraction increased after hematocrit reduction (Fig. 3, *F* and *G*).

##### Acid-base status.

$\text{P}{\text{a}}_{{\text{CO}}_{2}}$ at peak exercise remained unaltered after IVHD, whereas pH_a_ increased to non-EE values (Fig. 4, *A* and *B*). pH_v_ and $\text{P}{\text{v}}_{\text{C}{\text{O}}_{2}}$ tended to decrease after IVHD (Fig. 4, *C* and *D*), whereas BE and [lactate]_a_ remained statistically not different to pre-IVHD values (Fig. 4, *E* and *F*).

#### Non-EE versus EE post-IVHD.

##### Peak V̇o_2_ and O_2_ transport from air to arterial blood.

There were no differences between non-EE and EE post-IVHD in peak V̇o_2_ or V̇e (Fig. 2, *A* and *B*). $\text{P}{\text{a}}_{{\text{O}}_{2}}$, $\text{S}{\text{a}}_{{\text{O}}_{2}}$, and $\text{C}{\text{a}}_{{\text{O}}_{2}}$ were also similar between groups (Fig. 2, *C–E*). Dl and A-aPo_2_ were similar between groups (Fig. 2, *F* and *G*). Peak power output was not different between non-EE and EE post-IVHD groups (150.4 ± 13.4 vs. 140.5 ± 13.1 W, *P* = 0.19).

##### Peak Q̇t, SV, HR, and O_2_ transport from arterial blood to muscle.

After IVHD, peak Q̇t increased in highlanders with EE matching non-EE values (Fig. 3*A*), whereas peak Q̇o_2_ was slightly diminished (Fig. 3*B*). SV was not different in participants without EE and those of the EE group post-IVHD (1.60 ± 0.32 vs. 1.40 ± 0.32 mL/kg, respectively, *P* = 0.26) in the same way as HR (165 ± 10 vs. 155 ± 20 beats/min, respectively, *P* = 0.26). ${\text{Pv}}_{{\text{O}}_{2}}$, $Sv_{O_{2}}$, $\text{C}{\text{v}}_{{\text{O}}_{2}}$, Dm, and O_2_ extraction were not different between groups (Fig. 3, *C–G*, respectively).

##### Acid-base status.

After IVHD, $\text{P}{\text{a}}_{{\text{CO}}_{2}}$ remained not different in the EE compared with the non-EE group (Fig. 4*A*), whereas $\text{P}{\text{v}}_{\text{C}{\text{O}}_{2}}$ decreased to match non-EE values (Fig. 4*C*). pH_a_, pH_v_, BE, and [lactate]_a_ at peak exercise were not different in EE compared with non-EE values (Fig. 4, *B*, *D*, *E*, and *F*, respectively).

In summary, our results show that V̇o_2_, V̇e, and pulmonary gas exchange at peak exercise were not different in highlanders with and without EE and were unaffected after IVHD despite lower $\text{C}{\text{a}}_{{\text{O}}_{2}}$ and Q̇o_2_ due to reduced [Hb] (Fig. 2). Peak Q̇t was lower in EE compared with non-EE highlanders and improved after IVHD. Although Q̇o_2_ decreased due to lower [Hb], this was balanced by higher O_2_ extraction, sustaining peak V̇o_2_ as shown in Fig. 3 Acid-base status and lactate concentration showed marginal or no differences between groups at peak exercise and were minimally affected by IVHD (Fig. 4).

##### Individual associations between O_2_ transport variables.

Pre-IVHD arterial [Hb] correlated inversely with peak Q̇t (*r* = −0.60, *p* = 0.02) but showed no correlation with peak V̇o_2_, V̇e, D_L_, A-aPo_2_, Dm, BE, $\text{P}{\text{a}}_{{\text{O}}_{2}}$, nor $\text{P}{\text{a}}_{{\text{CO}}_{2}}$. [Hb] did not correlate with any variable after IVHD.

**Figure 5.**
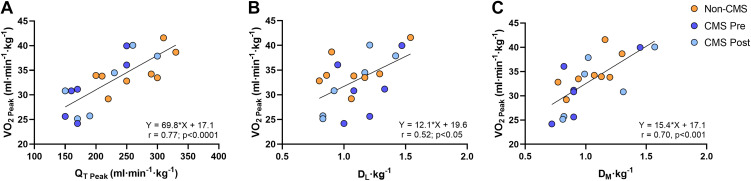
Components of oxygen transport associated with exercise capacity. Significant associations were observed between Q̇t (*A*, *P* < 0.0001), Dl (*B*, *P* < 0.05), and Dm (*C*, *P* < 0.001) with peak V̇o_2_. All variables measured at peak exercise and corrected by body weight. All male participants, non-EE (*n* = 8), EE (*n* = 6). CMS, chronic mountain sickness; EE, excessive erythrocytosis.

Before and after IVHD, peak V̇o_2_ showed a positive and strong association with peak Dl, Q̇t, and Dm (Fig. 5), but not with V̇e, A-aPo_2_, BE, $\text{P}{\text{a}}_{{\text{O}}_{2}}$, $\text{P}{\text{a}}_{{\text{CO}}_{2}}$, nor age. Similarly, V̇o_2_ did not correlate with BV nor its components (PV or total RBC volume) in our sample.

Among the significant associations between measured O_2_ transport variables, the slopes of the relationship between V̇o_2_ and Q̇t in non-EE and EE pre- and post-IVHD groups during the progression to peak exercise illustrate a slight improvement in Q̇t post-IVHD. The slope of the relationship is lower in participants with EE before IVHD relative to those without (4.2 ± 0.9 vs. 6.0 ± 1.0, *P* = 0.001) and tended to increase after IVHD, whereby it is no longer different compared with the non-EE group slope (5.2 ± 1.2 vs. 6.0 ± 1.0, *P* = 0.14) (see appendix).

### Multivariate Analysis of V̇o_2_ and O_2_ Transport Variables at Peak Exercise

Each of the univariate significant predictors (Dl, Q̇t, Dm) of peak V̇o_2_ was tested and included in multivariate regression models. Dl and Dm (which did not exhibit a significant correlation with each other across participants) explained the majority of variation in peak V̇o_2_, whereas Q̇T did not explain any of the variation in peak V̇o_2_ (Table 3).

**Table 3.** Multivariate linear regression models for V̇o_2Peak_

Independent Variable*	Coefficient of Regression (β)	SE	*P* Value	Model *R*^2^	Model *P* Value
*Pre-IVHD*					
Dl	11.63	3.67	0.010	0.89	<0.0001
Q̇t	9.89	14.92	0.52		
Dm	12.50	2.76	0.001		
Intercept	5.29	3.33	0.14		
*Post-IVHD*					
Dl	12.60	4.83	0.026	0.91	<0.0001
Q̇t	17.75	17.11	0.32		
Dm	10.50	2.12	0.0006		
Intercept	4.099	3.108	0.22		

*All variables at peak exercise and corrected by body weight. IVHD, isovolumic hemodilution.

## DISCUSSION

Our results show that at 4,340 m, Andean highlanders with EE have similar exercise capacity as their non-EE counterparts, and that peak V̇o_2_ is unaffected by IVHD in participants with EE, despite a significant reduction of arterial O_2_ concentration. In addition, the considerable variance in peak V̇o_2_ we noted across participants is not determined by the presence or absence of EE. Rather, we found that variance in peak V̇o_2_ was closely related to individual differences in lung and muscle diffusing capacity that drive the diffusive components of O_2_ transport.

### Isovolumic Hemodilution

Before IVHD, PV was not different between groups, supporting the concept that higher hematocrits are mainly the result of larger RBC total volumes in Andeans (29). The degree of reduction of hematocrit and [Hb] sought in this study was similar to the average decrease obtained in the few previous IVHD studies. As expected, CMS symptoms resolved rapidly within 24–48 h after hemodilution without signs of improved systemic blood oxygenation at rest through pulse oximetry or direct $\text{P}{\text{a}}_{{\text{O}}_{2}}$ measurement. Although alleviation of symptoms is a universal finding after hemodilution or bloodletting in CMS highlanders, and hence the most common management strategy for the syndrome, improved SpO_2_ or $\text{P}{\text{a}}_{{\text{O}}_{2}}$ is not observed in all individuals (22–25). In a study of six CMS highlanders in La Paz, Bolivia (3,650 m), Manier et al. in their study showed that at rest, $\text{P}{\text{a}}_{{\text{O}}_{2}}$, ${\text{Pv}}_{{\text{O}}_{2}}$, and A-aPo_2_ did not change significantly after IVHD. However, they did observe an increase in Q̇t and V̇e together with a slight but consistent improvement in V̇a/Q̇ mismatching, which could have resulted from the increase in blood flow perfusing poorly ventilated lung areas.

Our study confirms the findings of unchanged $\text{P}{\text{a}}_{{\text{O}}_{2}}$ and does not show any modification in Q̇t nor V̇e at rest after IVHD. However, it should be noted that our post-IVHD measurements occurred ∼48 h after the procedure, whereas measurements in the aforementioned study took place on the same day. The study from La Paz suggested that the increase in Q̇t resulted from a fall in blood viscosity due to an effect in main venous stems where shear rates are low. The fall in resistance increased venous return and led to an increase in ventricular filling, stroke volume, and HR, causing a rise in Q̇t and a concomitant fall in pulmonary vascular resistance. A more recent study on IVHD in 11 CMS highlanders in Cerro de Pasco undergoing a similar reduction of hematocrit showed an increase in Q̇t and PASP measured 1 day after IVHD (26) with hematocrit and Q̇t remaining stable at 14 days. A progressive development of iron deficiency (indicated by a 66% reduction in ferritin) was accompanied by a further significant increase in PASP ∼25% from baseline. Although similar in timeframe, we did not observe any change in Q̇t at rest after 48 h in our study. Although we did observe a significant reduction of serum iron and ferritin, we cannot confirm an increase in pulmonary artery pressure (PAP) as this was not measured. However, we cannot rule out a possible PAP-mediated limitation/restraint of further increase in peak Q̇t after IVHD, which might have required more than a modest increase to improve peak V̇o_2_ significantly.

### Exercise Capacity, Cardiac Output, Lung and Muscle Diffusing Capacity

The similarity of peak V̇o_2_ in highlanders with excessive and nonexcessive high-altitude hematocrits suggests a counterbalancing effect of elevated hematocrit and [Hb] on exercise capacity at altitude, whereby the gains in $\text{C}{\text{a}}_{{\text{O}}_{2}}$ afforded by the higher [Hb] in EE are offset by reductions in another step of the O_2_ transport cascade, in this case, Q̇t, such that arterial O_2_ delivery at peak exercise remained equal to that in participants without EE. In addition, some Andean highlanders with EE appear to have a well-adapted vascular phenotype (large arteries to normalize shear stress, and tight adrenergic control of the expanded vasculature) (48) that might counteract increased blood volume and viscosity. Similarly, the preserved peak V̇o_2_ (either absolute or corrected by body weight) after IVHD was also the result of counterbalancing effects of different steps of the O_2_ pathway—here a decrease in Q̇o_2_ balanced by an increase in O_2_ extraction. To better understand these counterbalancing effects, it is necessary to explore not just the differences among groups in each step, but also the interactions among the different steps by analyzing each of these through a systems approach.

#### Non-EE versus EE pre-IVHD.

Our step-by-step O_2_ transport analysis indicates that before IVHD, highlanders with EE had similar V̇e, $\text{P}{\text{a}}_{{\text{O}}_{2}}$, $\text{S}{\text{a}}_{{\text{O}}_{2}}$, lower Q̇t but sustained peak V̇o_2_ due to increased [Hb], $\text{C}{\text{a}}_{{\text{O}}_{2}}$, and hence similar Q̇o_2_ compared with participants without EE (Table 2). In terms of the compound constant D/(β·Q̇t), highlanders with and without EE sustain diffusional conductance in lungs and muscle and attain similar peak V̇o_2_ by counterbalancing the product β·Q̇t. Both groups have similar Dl and Dm values (Fig. 2*F* and Fig. 3*F*); however, participants without EE have lower β due to lower [Hb] and higher Q̇t, whereas highlanders with EE have higher β and lower Q̇t. This results in similar O_2_ diffusion between alveolar gas and capillary blood in the lungs and between muscle microcirculatory vessels and the mitochondria, as indicated by similar A-aPo_2_ and O_2_ extraction among groups (Fig. 2*G* and Fig. 3G). This similarity indicates that the slightly lower P_50 STD_ in highlanders with EE did not contribute significantly to facilitate O_2_ uptake in the lung capillaries or hinder O_2_ extraction in muscle.

#### EE pre-IVHD versus EE post-IVHD.

After IVHD, although the reduction in [Hb] should have affected O_2_ diffusion both in lungs and muscle, our results show that peak V̇o_2_ in the EE group is mainly maintained by an increase in the ability to move O_2_ by diffusion between microcirculatory vessels and muscle, and not between alveolar gas and capillary blood in the lungs (as indicated by an unchanged A-aPo_2_, Fig. 2*G*), but due to a greater post-IVHD O_2_ extraction (Fig. 3*G*). The significant reduction in [Hb], and hence in β, might have completely offset the impact of the slight increase in Q̇t in the compound constant. Despite post-IVHD iron depletion and its potential effect in having increased PAP and affected pulmonary capillary blood volume during our study, we found similar Dl values in participants without and with EE before hemodilution, with similar iron and ferritin values, and after hemodilution with significant iron depletion. Thus, we consider it unlikely that iron depletion could have affected pulmonary O_2_ diffusion.

Given that Dl and Dm remained unchanged after IVHD (Fig. 2*F* and Fig. 3*F*), the differential effect on diffusional conductance in lungs and muscle might be explained by a slightly increased vasoconstrictor effect (31) which restrains vasodilation within the active skeletal muscle slowing down local blood flow [further increasing Dm/(β·Q̇t)] and allowing more time for diffusional unloading of O_2_, thus contributing to sustain aerobic capacity (48).

The slightly reduced arterial [lactate] at peak effort after IVHD supports the idea of improved aerobic metabolism due to improved O_2_ diffusion and increased O_2_ extraction. Increased O_2_ extraction is also reflected in the slight ${\text{Pv}}_{{\text{O}}_{2}}$ reduction in the face of unchanged $\text{P}{\text{a}}_{{\text{O}}_{2}}$. Surprisingly, although $\text{P}{\text{a}}_{{\text{O}}_{2}}$ was similar to the pre-IVHD condition, $\text{S}{\text{a}}_{{\text{O}}_{2}}$ increased at peak exercise after IVHD possibly as a consequence of a relative metabolic alkalosis that causes a left shift of the O_2_-Hb dissociation curve. It is possible that the colloid plasma-replacement substance may have caused a transient increase in strong-ion difference resulting in the observed relative metabolic alkalosis (49). Although we did not measure renal function, the maintenance of this relative alkalosis could have been influenced by kidney hypoperfusion due to the slight BV reduction compared with the pre-IVHD condition. Due to the increase in plasma volume after IVHD, we can rule out increased bicarbonate concentration due to a contracted extracellular fluid compartment (50, 51). Although we observed a drop in body weight, which has been previously associated to increased diuresis after IVHD due to increased renal blood flow, effective plasma flow, or filtration fraction (22), we believe that this reduction could also relate to the dramatic decrease in total RBC mass. Considering an average decrease of 30 mL/kg in RBC mass from the pre- to the post-IVHD condition, and taking into account the specific gravity of a volume of RBCs (1.11 g/ml (52), the reduction of RBC mass results in 33.3 g RBCs/kg. If we consider the average body weight of participants, the total decrease of RBC mass is ∼2.4 kg, which is comparable with the observed decrease in average body weight. Unfortunately, we cannot determine the direct cause of body weight since we did not measure daily water intake nor water excretion.

Finally, the slightly higher P_50 STD_ after IVHD may be also a small contributing factor to the higher O_2_ extraction. In terms of the Dm/(β·Q̇t) constant, an increase in P_50_ (right shift of the O_2_-Hb dissociation curve) has the effect of making β smaller, increasing Dm/(β·Q̇t), and improving extraction. However, any small effect of higher P_50 STD_ on O_2_ transport would have been offset by a greater effect of the relative metabolic alkalosis, so the net result was still a leftward shift of the curve.

#### Non-EE versus EE post-IVHD.

After IVHD, the participants with EE maintained similar peak V̇o_2_ compared with those without (Fig. 2*A*), and most variables that were different in the group with EE before IVHD turned similar to non-EE after hematocrit reduction. Q̇t increased slightly (Fig. 3*A*) and [Hb] decreased to non-EE levels (Fig. 1*E*), which in terms of the compound constant D/(β·Q̇) would have equalized O_2_ diffusion at the lungs and muscle given the similarity on Dl and Dm between groups (Fig. 2*F* and Fig. 3*F*). We observed no difference in the A-aPo_2_ and, although after IVHD Q̇o_2_ was slightly reduced in EE compared with non-EE (Fig. 3*B*), the trends for lower ${\text{Pv}}_{{\text{O}}_{2}}$, $Sv_{O_{2}}$ (at similar pH_v_, Fig. 3, *C* and *D*, and Fig. 4*D*, respectively) and higher O_2_ extraction indicate that peak V̇o_2_ is mainly maintained by O_2_ diffusion at the working muscle level.

### Causes of Variance in Peak V̇o_2_ across Participants

Despite no differences in peak V̇o_2_ between non-EE and EE groups, either before or after IVHD, findings which answer our primary question in this study, there was considerable (not quite twofold) variation in peak V̇o_2_ across all participants which was unrelated to age or [Hb] in our sample. We therefore sought to determine whether this variance could be explained by one or more steps in the O_2_ transport pathway and approached this by multivariate linear regression analysis.

First, univariate regressions across all participants (with and without EE) showed significant positive correlations between peak V̇o_2_ and peak Q̇t, Dl, and Dm. Second, multivariate analysis showed significant contributions only from Dl and Dm, suggesting that, independently of Q̇t, differences in diffusing capacities of the lung and muscle are the main determinants of variance in aerobic capacity in Andean men at altitude, whether they have EE or not (Table 3).

That the two diffusive steps of the O_2_ pathway are the main determinants of variation in peak V̇o_2_ at altitude makes inherent sense because the convective steps involving ventilation and cardiac function are not diminished but, on the contrary, unaffected or enhanced by altitude. This has been shown even when hypoxia is as extreme as that found on the Everest summit (53–55). However, the diffusive movement of O_2_, both from alveolar gas into blood and from blood to muscle mitochondria, is the product of the relevant diffusing capacities and the Po_2_ gradient in each location. Altitude clearly lowers the Po_2_ gradients in both lung and muscle, making diffusive movement of O_2_ more dependent on values for Dm and Dl.

### Limitations

One limitation of our study is that all participants were adult males, generally of middle age, and thus results and conclusions should therefore be limited to the sex and age group included. Also, we cannot rule out a possible effect of variance in the number of years participants had EE and thus had been exposed to an excessive hematocrit before our study. The longer an individual lives with an excessive hematocrit, the greater is the potential for vascular and hemodynamic impact/damage, which perhaps cannot be reversed acutely with hemodilution. Unfortunately, it was not possible to obtain precise information regarding the length of exposure to EE of our participants. We believe that these limitations do not invalidate the conclusions of our study since logically consistent differences in each O_2_ transport variable assessed were observed between groups despite using a battery of independent measurements (expired gas analysis, impedance cardiography, and arterial blood sampling). Moreover, each participant with EE was his own control (comparing pre-IVHD with post-IVHD states), and the results were consistent in each of them. Also, although only ∼90% of V̇o_2_ at peak exercise is attributable to working muscle, the conclusions made from measured or calculated whole body variables (which use whole body V̇o_2_ measured from expired gas analysis to reflect the working muscles) maintain validity based on the same data treatment for each group and also on the criteria of differences between groups, rather than in absolute numbers.

A possible additional limitation to our study is the contribution of age to the variation in peak V̇o_2_. Analysis of pooled data from our study and two different studies in male highlanders from Cerro de Pasco in the age range 24–64 yr (30, 56) shows a weak but significant correlation between peak V̇o_2_ and age (*r* = 0.33, *P* < 0.05, *n* = 43). Although we did not observe this association in our sample, possibly due the reduced number of participants, age was not an intrastudy determinant of peak V̇o_2_ neither in the non-EE nor the EE group both pre- or post-IVHD (either separate or combined), and thus can be considered as a uniform variable. Also, older participants did not show greater or lesser improvement of peak V̇o_2_ after IVHD.

Although we cannot rule out a possible limitation of increase in peak V̇o_2_ after IVHD due to the slight decrease in BV (allowing further increase in Q̇t), we consider that the post-IVHD preservation of peak V̇o_2_ despite the dramatic decrease in $\text{C}{\text{a}}_{{\text{O}}_{2}}$ remains the key finding of our study maintaining exercise capacity similar to that observed in healthy highlanders. Finally, the drop in body weight could be considered as a potential source of variation of peak V̇o_2_ and in the interpretation of results. However, we found no difference in the absolute peak V̇o_2_ measurements (mL·min^−1^) after IVHD, and if any, the effect of body weight reduction would have resulted in an increase in relative peak V̇o_2_ (mL·min^−1^·kg^−1^), which we did not observe in any case.

### Conclusions

In conclusion, we find that when tested at their resident altitude of 4,340 m, Andean men with EE have the same exercise capacity as male Andeans of the same age without EE. This is explained by lower Q̇t balancing the higher arterial [O_2_], equalizing systemic O_2_ delivery, combined with similar O_2_ extraction. Furthermore, we find that acute reduction in hematocrit by 20% using isovolumic hemodilution does not alter peak exercise capacity, despite reducing arterial O_2_ concentration and systemic O_2_ delivery. Here, peak V̇o_2_ is preserved by greater O_2_ extraction. Despite group similarities in peak V̇o_2_, there is an almost twofold variance in exercise capacity across all participants. This was explained essentially completely by differences in both pulmonary and muscle O_2_ diffusional conductances and not by any differences in pulmonary ventilation, [Hb], nor Q̇t.

## GRANTS

F.C.V. is supported by a Wellcome Trust grant 107544/Z/15/Z. T.S.S. is supported by the National Institutes of Health R01HL145470, the National Geographic Society Explorer Award, and the John B. West Endowed Chair in Respiratory Physiology.

## DISCLOSURES

The authors declare that the research was conducted in the absence of any commercial or financial relationships that could be construed as a potential conflict of interest.

## AUTHOR CONTRIBUTIONS

P.D.W., T.S.S., and F.C.V. conceived and designed research; C.A.-R., J.L.M., R.J.F.-M, G.A.V.-G., E.C.H., M.S.T., H.E.W., P.D.W., T.S.S., and F.C.V. performed experiments; C.A.-R., W.G., R.J.F.-M., G.A.V.-G., P.D.W., T.S.S., and F.C.V. analyzed data; W.G., P.D.W., T.S.S., and F.C.V. interpreted results of experiments; W.G. and F.C.V. prepared figures; P.D.W., T.S.S., and F.C.V. drafted manuscript; C.A.-R., W.G., J.L.M., R.J.F.-M., G.A.V.-G., E.C.H., M.T., H.E.W., P.D.W., T.S.S., and F.C.V. edited and revised manuscript; C.A.-R., W.G., J.L.M., R.J.F.-M., G.A.V.-G., E.C.H., M.T., H.E.W., P.D.W., T.S.S., and F.C.V. approved final version of manuscript.

## Figures and Tables

**Figure A1.**
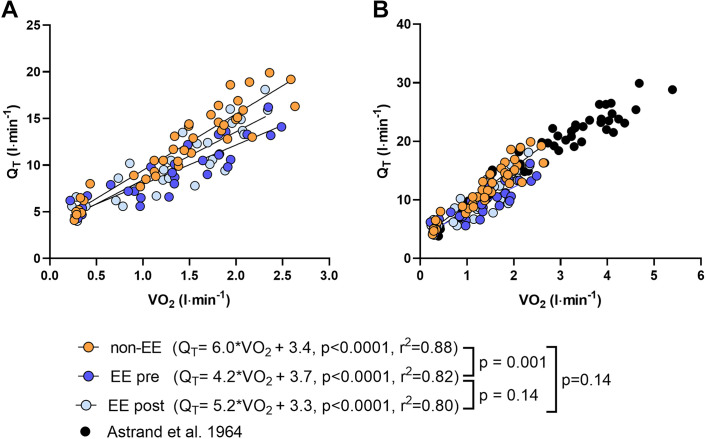
Relationship between Q̇t and V̇o_2_ at rest and during the different stages of incremental exercise. *A*: Q̇t and V̇o_2_ showed a strong positive correlation in the three groups throughout incremental exercise. The regression slope was shallower in the group with EE compared with the group without EE. After IVHD, the slopes were no longer different due a steepening of the slope in the group with EE. Differences between the slopes of the three regressions were assessed by ANOVA. Significance established at *P* < 0.05; non-EE vs. EE pre: *P* = 0.001, EE pre vs. EE post: *P* = 0.14, non-EE vs. EE post: *P* = 0.14. All male participants, non-EE (*n* = 8), EE (*n* = 6). *B* shows how values from all three groups overlay with data obtained from the classic study by Astrand et al. (43). Our measurements in participants without EE agree well with the data from Astrand et al., whereas measurements in participants with EE show slightly lower values for Q̇t at any exercise intensity before IVHD, returning to the Astrand relationship after IVHD. EE, excessive erythrocytosis; IVHD, isovolumic hemodilution.

## APPENDIX

The classic study from Astrand and collaborators from 1964 (43) used the dye (indocyanine green) dilution technique to estimate Q̇t. Our Q̇t data obtained using impedance cardiography (IC) from participants without EE superimposes almost perfectly to the Astrand et al. study (Fig. A1), whereas participants with EE show slightly lower values for Q̇t at any exercise intensity that might result from the effect of increased blood viscosity in main venous stems, and slightly decreased venous return, decreased ventricular filling, and SV.
